# Editorial: The changed life: how COVID-19 affected people's psychological well-being, feelings, thoughts, behavior, relations, language and communication

**DOI:** 10.3389/fpsyg.2023.1285573

**Published:** 2023-10-05

**Authors:** Ramona Bongelli, Alessandra Fermani, Daniela Raccanello, Rob Hall, Ilaria Riccioni, Morena Muzi, Roberto Burro

**Affiliations:** ^1^Department of Political Science, Communication and International Relations, University of Macerata, Macerata, Italy; ^2^Department of Education, Cultural Heritage and Tourism, University of Macerata, Macerata, Italy; ^3^Department of Human Sciences, University of Verona, Verona, Italy; ^4^Department of Psychology, Macquarie University, Sydney, NSW, Australia

**Keywords:** COVID-19, long psychological effects, coping, emotional distress, health, anxiety, feelings, resilience

## 1. Introduction

The COVID-19 pandemic impacted deeply on the lives of millions of people around the world. In addition to the effects of the virus on physical health, the pandemic also had considerable psychological and social effects on individuals, the outcomes of which, just like the physical ones, are slow to disappear.

Thus, the expression “long-COVID” seemed to be applicable not only to describe the long-term effects on the physical health of people who have contracted the virus, but also to refer to the long-term psychological outcomes, identifiable both in those who have been infected and, more generally, in all those who had to cope with the loss of relatives and friends, the fear of being infected and/or infecting others, as well as with social restrictions, lockdown, distancing, working and studying remotely, and other similar challenges.

The way of handling these difficulties has been very different and, while there were people who were overwhelmed by fear, uncertainty, anxiety, and depression, there were others who have shown remarkable resilience.

## 2. The Research Topic

In the context of a pandemic from which—at least in medical terms—the world was slowly emerging, in May 2022 we launched our Research Topic entitled “The changed life: how COVID-19 affected people's psychological well-being, feelings, thoughts, behavior, relations, language and communication.”

In response to the call, we received many highly interesting papers and 42 of them (34 original research papers, 2 brief research reports, 4 reviews, 1 opinion paper, and 1 empirical study), authored by 215 researchers from 27 different countries, were successfully published: 36 in *Frontiers in Psychology* (sections Health Psychology, Education, Positive Psychology, Personality and Social Psychology, Pediatric Psychology, Quantitative Psychology and Measurement), 5 in *Frontiers in Education* (section Educational Psychology), and 1 in *Frontiers in Aging Neuroscience* (section Neurocognitive Aging and Behaviors).

The papers in this Research Topic cover a wide range of topics. Although the majority focuses on the negative psychological effects of the pandemic [such as increased anxiety, depression, fear, powerless, post-traumatic stress disorder (PTSD), burnout, emotion dysregulation (ED)], as well as on individuals' dysfunctional behaviors (e.g., excessive hoarding behavior, academic procrastination, epidemic information-avoidance behavior, nomophobia, and smartphone addiction), other papers focus on personal characteristics (e.g., specific traits of personality, resilience, and psychological flexibility) and behavioral habitus (e.g., performing physical exercise, participating in artistic and cultural activities, using social media to access medical information, and resorting to positive coping strategies), which seem to help people live more adaptively and functionally in a highly stressful situation, suggesting that the pandemic have been also a time of opportunity for personal growth and development. Lastly, some other papers specifically focus on distance learning, and more generally, on the use of online environments and social media.

Thus, despite the diversity of the contributions, they appear to fall within three main areas of research:

1. Psychological negative outcomes;
2. Resilience, post-traumatic growth, and adoption of functional coping strategies;
3. Distance learning, online environment, and social media.

### 2.1. Psychological negative outcomes

A first line of research includes papers examining the negative psychological outcomes of the pandemic. Indeed, as previously mentioned, a significant portion of the studies featured in this dedicated issue has mainly focused on the negative psychological and behavioral consequences resulting from the COVID-19 pandemic across diverse socio-demographic groups.

For instance, the study conducted by Park and Park drew attention to the impact of the pandemic on the quality of life and the manifestation of depressive symptoms among Korean patients coping with chronic illnesses. In a similar vein, Mao et al. delved into the influence of COVID-19-related fear among a cohort of Chinese individuals diagnosed with cancer. The findings of their investigation revealed that the level of fear associated with COVID-19 exhibited an inverse correlation with quality of life, while concurrently displaying a positive correlation with safety-conscious behavior.

The impact of COVID-19 affected patients and medical students' mental wellbeing (see for example, Abdolkarimi et al.) and healthcare practitioners. This is evident from the systematic meta-analyses carried out by Paz et al. and Tong et al., which showed elevated levels of stress, anxiety, and depression within these two distinct groups.

COVID-19 has had negative psychological consequences not only on medical students but more generally, on all students, especially those with specific personality traits. For example, in a study carried out by Zhang Y. et al., involving a group of 2,485 Chinese university students, the authors brought to light that individuals characterized by alexithymic tendencies and lower self-esteem were more prone to manifest symptoms indicative of PTSD as well as to encounter sleep-related issues. The adverse impacts of COVID-19 on the student population appear to transcend merely their current mental wellbeing, also encompassing their capacity to envision their future. The anxiety experienced by students appears to exert an adverse influence on their confidence regarding employment prospects and career expectations (Nazir and Özçiçek; Zhao; Zheng et al.).

Even teachers were not immune from the consequences of COVID-19 either (Weiher et al.). A considerable number of them grappled with the imbalance between the demands of their work and the available resources within the educational setting. The findings of a study undertaken by Rastegar and Raimi underscored the significance of avoidant strategies in detrimentally affecting teachers' overall wellbeing, while highlighting the role of problem-focused strategies in fostering enhancements in their mental health.

Chen et al. instead attempted to identify some social variables responsible for the negative impact of COVID-19 on adolescents' mental health, identifying them in the reduction of social trust and in the increase in inequality caused by the digital and economic divide. Also among the younger people the situation has been very complex. The main results of a cross-sectional analysis of quality of life and loneliness among United States (US) children early in the COVID-19 pandemic (Skeens et al.) revealed that children experienced a worse quality of life and a greater loneliness when compared to normative samples. These outcomes were worse for girls and older children and raise concern for short- and potentially long-term mental health sequelae due to the pandemic. The impact of the pandemic on children was partially explained by the distress related to COVID-19 experienced by parents. While social interactions with friends did not mitigate this indirect impact, enhanced family functionality appeared to offer a protective effect.

Other research has investigated the effects of COVID-19 and lockdown on the general population. Covelli et al., for example, examining an Italian sample, found that participants reported lower levels of general wellbeing and a higher level of both perceived stress and fear related to COVID-19 during the first lockdown period than at the time of the survey (a month after the first wave). In other terms, participants were observed to be enhancing their overall sense of wellbeing 1 month after the initial wave of the pandemic. Nonetheless, the persistence of fear and emotional ambivalence suggests paying attention to the sense-making of that event in order to support the correct reconstruction of experiences and emotions experienced in that period. Camisasca et al., in a sample of Italian women, found that the marital dissatisfaction experienced during the pandemic explains the indirect effects of economic difficulties on psychological maladjustment.

Within the spectrum of COVID-19-related negative behaviors, the contributors of this Research Topic identified positive correlations between fear of COVID-19 and hoarding tendencies (Zhao Y. et al.). Additionally, a correlation has been noticed between the perception of epidemic risk and the adoption of information avoidance behavior (Zhang K. et al.). On the contrary, anxiety seems to correlate positively with an excessive reassurance-seeking tendency (that can be considered as a form of maladaptive coping strategy), which was measured by Manrique-Millones et al. through the Coronavirus Reassurance Seeking Behavior Scale among a sample of 661 Peruvian adults. Obviously, many other dysfunctional behaviors were observed during the pandemic period. Among these there is a high alcohol consumption (Šulejová et al.).

### 2.2. Resilience, post-traumatic growth, and functional coping strategies

A second line of research includes papers focusing on psychological resilience, post-traumatic growth, as well as on the use of functional coping strategies.

Matsumoto et al., for example, conducted a study on changes in psychological resilience among a sample of 130 older adults with mild cognitive impairment (MCI) during the stressful period related to the COVID-19 pandemic. The results of their research show that the improvement in psychological resilience was associated with a good sleep quality. In other words, the better you sleep, the more resilient you are.

Marashi and Heisz found, instead, that resilience and physical activity (as for the protective role of physical activity, see also Drole et al.; Zhao W. et al.) seem to be protective factors against symptoms of anxiety and depression in graduate students experiencing increased academic stress during COVID-19. Analogously Reilly et al., in a sample of US military veterans, found that psychological flexibility, a process of modifiable resilience, buffered some of the negative impacts of the pandemic on mental health and quality of life.

Paoletti et al., following a shared reflection with resilience researchers—convinced that the pandemic can be a catalyst for change in building more resilient communities and social structures—identified and discussed in their paper four interdisciplinary lessons that COVID-19 can teach: (1) Being more aware of brain functioning and its potential can help us face the global increase in anxiety and depression; (2) It is necessary to develop an awareness of human interconnectedness to overcome adversity; (3) School-programs should educate next generations in resilience; (4) Self-training resilience tools can allow individuals, groups, and communities to access neuro-psycho-pedagogical knowledge to face adversities, uncertainty, and changes in everyday life.

As regards post-traumatic growth, the results emerging from Hao et al. study show interesting insights. The authors undertook an empirical investigation involving a cohort of 2,990 volunteer university students, enrolled at various universities and actively engaged in the containment and management of the epidemic situation. According to their findings, the adoption of a positive coping style and the perception of robust social support among university student volunteers showed a positive association with higher levels of post-traumatic growth. Conversely, the manifestation of a negative coping style showed a positive relationship with the amplification in the severity of PTSD symptoms experienced by these volunteers.

The role of social support has been also investigated by Dhruve et al.. Specifically, the authors explored ED and perceived social support (PSS) as potential mechanisms for the relation between COVID-19 stress and depressive symptoms among a sample of 489 students at a Southern university in the US. The results of their path analysis revealed that PSS buffered the effect of ED on depressive symptoms, suggesting that the perceived social connection may be an essential factor for psychological outcomes during periods of stress and isolation, particularly for those reporting high ED.

Consistent with these findings, also the results of Li et al., who have carried out a study among a cohort of Chinese female undergraduate students in the field of liberal arts, highlighted the protective role of social support against the negative effects of the pandemic.

With regard to adaptive coping strategies, Burro et al. examined the role that personality traits can play in their promotion. Investigating a sample of 2,995 Italian university students in the early stages of the pandemic, their research showed that in reference to the four families of coping strategies (Burro et al., 2021) called Despair, Aversion, Proactivity, and Adjustment: (a) university students reacted more frequently using adaptive coping strategies (with Proactivity used more frequently than Adjustment) rather than maladaptive strategies (with Despair higher than Aversion); (b) Agreeableness, Conscientiousness, and Open-Mindedness clearly revealed their protective role; (c) these personality traits were generally significantly related with each of the four families of coping strategies, specifically negatively with Despair and Aversion (albeit with some exceptions, in which the direction of the links was in line with our hypotheses but the relations were not significant) and positively with Proactivity and Adjustment.

Staying within the realm of education and delving into the viewpoint of educators, Duong et al. similarly emphasized the significance of perceived support. The outcomes of their investigation indeed unveiled that the support provided by school leaders played a pivotal role in enhancing the occupational wellbeing of teachers amidst the challenges posed by the COVID-19 pandemic.

In the general population, the perception of positive or negative changes related to COVID-19 also seems to be linked, according to the results of Jurišová et al., to optimism, pessimism, and levels of hope experienced by individuals. David and Truta also found interesting associations between personological characteristics (dedicated type), on the one hand, and personal internal resources (e.g. creativity, playfulness, wellbeing, and personal meaning) and psychological wellbeing, on the other. According to the authors, in order to promote the psychological and mental health of individuals, it is necessary to provide interventions that elicit meaning, stimulate creativity, and guide people in their search for purpose.

### 2.3. Distance learning, online environment, and social media

A third line of research includes papers highlighting issues concerning distance learning and the use of social media as information tools. Specifically, the studies on distance learning have primarily focused on evaluating the relationships between, on the one hand, students' abilities to organize and plan learning or to procrastinate, that is, to postpone work on a task (e.g., Muarifah et al.) and, on the other hand, their academic performance (e.g., Xu et al.; Lv et al.) or the perceived quality of learning (e.g., Roberts et al.; Sergi et al.). A meta-analysis conducted by Xu and Xue, examining the views of parents, teachers, and students, showed the prevalence of satisfaction with online education. However, it was observed that the percentage of satisfied students was relatively lower than that of teachers and parents.

As far as social media and more in general online environments are concerned, although some research has highlighted, during the COVID-19 home confinement, the worsening of psychological addiction related to the use of smartphones, especially among adolescents (see, for example, Aydin and Kus), other research has, on the contrary, emphasized the functional use of social media, which can provide a broader access to health information, offer greater opportunities for health surveillance (Kim et al.), and may influence (along with the exposure to other media channels) health behaviors, especially in some older segments of the population (He et al.). Furthermore, other studies have shown how remote engagement provided important mental health support throughout the pandemic lockdown, although with limitations on feelings of social connectedness within online environments (Chapple et al.).

## 3. Conclusion

As evident from the provided summary, the articles featured in this Research Topic are very different in terms of the aims pursued, the samples under examination, the methodologies employed, and the outcomes observed. It is precisely this diversity that enriches their insights, contributing to drawing an exceptionally comprehensive overview of the pandemic outcomes. Aside from enhancing comprehension of the impacts of the COVID-19 pandemic on individuals' lives, these articles can also have practical implications, contributing to the identification of interventions geared toward enhancing mental wellbeing in the midst of, and following, catastrophic events like the COVID-19 pandemic. Governments should adopt public health policies that take into account not only the physical, but also the psychological, mental, emotional, and relational health of individuals.

## Author contributions

RB: Writing—original draft, Writing—review and editing. AF: Writing—original draft, Writing—review and editing. DR: Writing—original draft, Writing—review and editing. RH: Writing—original draft, Writing—review and editing. IR: Writing—original draft, Writing—review and editing. MM: Writing—original draft, Writing—review and editing. RB: Writing—original draft, Writing—review and editing.
